# Getting inside the engine - myosin modulation in hypertrophic cardiomyopathy and systolic heart failure

**DOI:** 10.1161/CIRCULATIONAHA.121.056324

**Published:** 2021-09-07

**Authors:** Matthew J Daniels, Luca Fusi, Christopher Semsarian, Srihari S Naidu

**Affiliations:** 1 Manchester Heart Centre, Manchester Royal Infirmary, Manchester University NHS Foundation Trust, UK; 2 Division of Cardiovascular Sciences, Manchester Academic Health Sciences Centre, University of Manchester, Manchester, UK; 3 Division of Cell Matrix Biology and Regenerative Medicine, University of Manchester, Manchester, UK; 4 Randall Centre for Cell and Molecular Biophysics and BHF Centre for Research Excellence, King’s College London, London, UK; 5 Agnes Ginges Centre for Molecular Cardiology at Centenary Institute, The University of Sydney, Australia; 6 Hypertrophic Cardiomyopathy Center, Department of Cardiology, Westchester Medical Center, Valhalla, NY, United States

**Keywords:** Hypertrophic Cardiomyopathy, Systolic Heart Failure, Myosin, Mavacamten, Omecamtiv

The palpable heartbeat requires synchronous activation, and inactivation, of trillions of motors, compartmentalised in billions of cardiomyocytes, in a fraction of a second. To bring the biophysical concepts of myocardial contraction, in health and disease, closer to the practicing cardiologist we draw parallels with motor vehicles to discuss both recent advances describing how the heart modulates itself and a new era of myocardial therapeutics.

Contraction requires two proteins, myosin and actin. Myosin (MYH7) is the molecular engine of the heart, converting the chemical energy of ATP into movement. Force production at the level of the cardiomyocyte occurs when millions of myosin engines combine, pulling each of ~50 sarcomeres per heart cell ~10% of their resting length closer (Figure 1A). Mechanical work changes rapidly, and reversibly. This is most obvious within a heartbeat, between systole (contraction) and diastole (relaxation). Additionally, the Frank-Starling mechanism adjusts performance beat-to-beat in response to changing pre- and after-load. Finally, cardiac adaptation to pregnancy, exercise, or injury develops over longer periods. Here we simplify what happens under the hood, and explain how this applies to patients.

The way myosin works dictates how the heart regulates force production. Myosin takes fixed steps (~10nm), that generate small amounts of force (picoNewton range), using an ATP hydrolysis mechanism that is slow (10s^-1^). Flexibility requires variable recruitment of myosin motors. Therein lies the challenge of regulation. If output is a numbers game, how do you pack a cell full of engines sufficient for a burst of activity but allow the whole system to idle most of the time? How do you hard-wire changes needed for prolonged elevations in activity like pregnancy, while retaining the ability to handle variations in pre-load that come from breathing? Distinct control mechanisms (clutches, gears and accelerators) are needed.

Actin is both the road that myosin drives on, and the stimulator of Myosin-ATPase activity that powers movement. The engine only burns fuel when it has road to run on. The 100-fold variation in ATPase activity between systole and diastole results from a molecular clutch under the control of calcium (Ca^2+^) housed in the thin filament troponin complex. This permits the interaction between motor and road in high systolic (1.0μM) calcium, but disengages it in low (0.1μM) diastolic calcium (Figure 1B).

Other inputs tune this, notably the adrenergic system, which directs phosphorylation of myofilament components, and changes intracellular calcium thresholds. Tuning adjustments take time, and are used when sustained changes (shifting gears) in contractility are required. These molecular gears ensure durable changes in cardiac output, but are too slow for beat-to-beat control [1].

## Myofilament-based regulation of cardiac contractility

**Figure 1:**
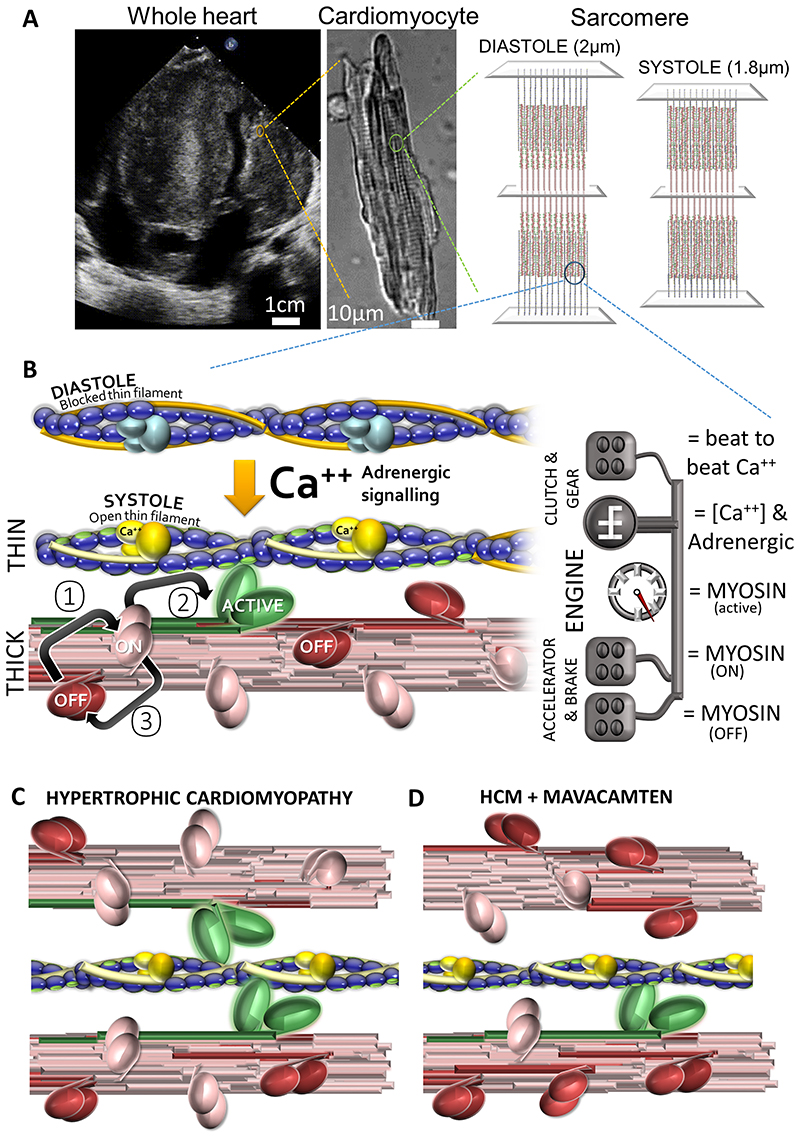
Myofilament activation and modulation. **(A)** Contractility of the whole heart, in this case a heart with hypertrophic cardiomyopathy and massive septal thickening, arises from the coordinated activation of myosin motors organised into regular sarcomere arrays in single cardiomyocytes. **(B)** Force production is controlled by regulating myosin activity. Actin stimulates the myosin ATPase. Actin availability is controlled by fluctuating calcium levels which via the troponin complex in the thin filament regulate a molecular clutch shielding actin (purple balls) from myosin in low diastolic calcium (Ca^++^) and exposing it in high systolic Ca^++^. Myosin is bundled, forming the thick filament. The exposed head contains the motor domain which cycles between “super-relaxed” OFF (red), “relaxed” ON (pink) and “Active” force producing states (green). In diastole the OFF state predominates. In systole as the myofilament activates, an intrinsic thick filament mechanism facilitates the transition between OFF and ON (①). Peak contractility occurs with 1:10 myosin heads active. Small molecule modulators of this system are in clinical evaluation. Omecamtiv mecarbil, a Myosin ATPase activator, increases force production by stimulating transition ②. Mavacamten, a myosin ATPase inhibitor, stabilises the super-relaxed OFF state ③. **(C)** Hypertrophic cardiomyopathy-causing variants typically reduce myosin locked in the OFF state. With more myosin active, force production in systole and diastole increases (more green, fewer red myosin heads). **(D)** Restoring the balance of myosin activation in HCM, using myosin ATPase inhibitors like mavacamten, reduces force production (and ATP consumption) in systole and diastole (more red, less green).

The molecular accelerator/brake which underpins the Frank-Starling mechanism is housed in myosin itself. Myosin cycles between OFF (cannot bind actin), ON (can bind actin) and ACTIVE states (Figure 1B). In diastole most of the 294 myosin motors in each half-sarcomere thick filament are in the OFF state. In systole, a fraction of these are recruited for contraction; typically, only ~30 motors per half filament bear the peak force [1]. Although only 10% of all motors are attached to actin at peak force, this does not mean that 90% do nothing. As ~400 ATP molecules per half filament are consumed during contraction, each motor probably undergoes one or two cycles per heartbeat [1]. Transitions between the ON and OFF states are influenced by Myosin Binding Protein C (MYBPC3), the myosin regulatory light chain (and kinase), and on a beat-to-beat basis by a mechanosensitive mechanism residing in the thick filament which enables force dependent recruitment of myosin heads from the OFF state [1].

## Diseases of the myofilament

Hypertrophic cardiomyopathy (HCM) is an inherited disease, typically due to pathogenic variants of the thick (~70%) and thin filament (~20%). HCM cardiomyocytes expend more energy in systole and diastole. Since ATP consumption/force production comes from myosin, genetic changes facilitating longer or stronger interactions with actin, or which encourage more myosin heads to participate in any given contractile cycle, could cause this.

Most HCM-causing variants increase the proportion of ACTIVE myosin (more accelerator, less brake), resulting in both diastolic dysfunction and hypercontractility (Figure 1C). *MYH7* pathogenic variants reduce how much myosin is sequestered into the OFF state, or reduce sensitivity to the molecular signal coming from MYBPC3 (less brake) which promotes this transition [2]. *MYBPC3* pathogenic variants by contrast exert less influence on MYH7 (reducing the proportion in the OFF state) or bring thick and thin filaments closer together promoting actin/myosin interaction. Conversely some thin filament mutations increase the amount of Ca^++^, and the duration it is held, in the sarcomere [3] triggering hyper-contractility through actin availability.

A subset of inherited dilated cardiomyopathy (DCM) is due to pathogenic variants in the same myofilament genes, but with opposite molecular effects on contractility.

## Direct pharmacological modulation of the myofilament in theory and practice

Established negative or positive inotropes target the engine indirectly via the gears, without delivering a prognostic impact in various conditions aside from the proven, yet counter-intuitive, role of beta-blockade in chronic systolic heart failure. Controlling the engine directly via the accelerator/brake may be clinically useful, is now biologically plausible, and potentially free from some of the undesirable effects of existing inotropes, such as effects on heart rate.

If most motors are idle, and only 10% needed for peak force, perhaps recruitment could be increased pharmacologically with clinical benefits in systolic heart failure? Conversely, in advanced systolic heart failure, if the control systems drive the engine to destruction, rather than push it harder, beta-blocker data might suggest a value of preventing the engine overheating by suppressing the motor? This hypothesis could be tested in HCM disease states which rev the engine continuously (Figure 1C), and indeed a number of small molecules targeting the myofilament are now developed and in clinical trials.

Several broad classes of myofilament therapeutics exist. Myosin activators include Omecamtiv mecarbil. The phase III GALACTIC-HF trial in chronic systolic heart failure reported a modest reduction in heart failure hospitalisations without reducing heart failure deaths, or improving quality of life [4], while the phase IIa study of Danicamtiv reported favourable echocardiographic remodelling. Myosin inhibitors include mavacamten (increases the proportion of myosin in the OFF state, Figure 1D). The phase III EXPLORER-HCM for symptomatic obstructive HCM reduced left ventricular outflow tract gradients, with improved heart failure biomarkers, symptoms, exercise performance, and health status [5] but with a narrow therapeutic window, and limited additional benefit in patients tolerating beta-blocker. Shorter half-life mavacamten-like molecules (e.g. MYK-581, MYK-224) are in early evaluation, the most progressed being CK-274, with a completed, but not yet reported phase II study. Calcium sensitizers and desensitizers that work on the clutch are also in development but fall outside the scope of this article.

## Summary

The contractile apparatus responds rapidly, and reversibly, to fluctuating pre- and after-load using molecular accelerators and brakes to control the engine at any particular gear. It is now possible to stimulate or suppress the engine directly with a novel class of myocardial therapeutics. This conceptual framework of clutch, gears, and accelerators or brakes should help clinicians understand and categorize future directions in sarcomeric therapies for systolic and diastolic dysfunction.
